# Etomidate and Ketamine: Residual Motor and Adrenal Dysfunction that Persist beyond Recovery from Loss of Righting Reflex in Rats

**DOI:** 10.3390/ph8010021

**Published:** 2014-12-29

**Authors:** Daniel Diaz-Gil, Noomi Mueller, Ingrid Moreno-Duarte, Hsin Lin, Cenk Ayata, Cristina Cusin, Joseph F. Cotten, Matthias Eikermann

**Affiliations:** 1 Department of Anesthesia, Critical Care and Pain Medicine, Massachusetts General Hospital, 55 Fruit Street, Boston, MA-02114, USA; E-Mails: diazgil.daniel@gmail.com (D.D.-G.); nmueller1@partners.org (N.M.); ingrid.md.8@gmail.com (I.M.-D); jcotten@mgh.harvard.edu (J.F.C.); 2 Department of Anesthesia, Critical Care and Pain Medicine and Department of Pharmacy, Massachusetts General Hospital, 55 Fruit Street, Boston, MA-02114, USA; E-Mail: hlin13@mgh.harvard.edu; 3 Department of Neurology, Massachusetts General Hospital, 55 Fruit Street, Boston, MA-02114, USA; E-Mail: cayata@mgh.harvard.edu; 4 Department of Psychiatry, Massachusetts General Hospital, 55 Fruit Street, Boston, MA-02114, USA; E-Mail: ccusin@mgh.harvard.edu; 5 Department of Anesthesia Critical Care and Pain Medicine, Massachusetts General Hospital, 55 Fruit Street, Boston, MA-02114 and Klinik fuer Anaesthesie und Intensivmedizin, Universitaetsklinikum Essen, 45147 Essen, Germany

**Keywords:** etomidate, ketamine, anesthesia, hyperlocomotion, coordination, adrenal function

## Abstract

We tested the hypothesis that etomidate and ketamine produce residual effects that modify functional mobility (measured by the balance beam test) and adrenal function (adrenocorticotropic hormone (ACTH) stimulation) immediately following recovery from loss of righting reflex in rats. Intravenous etomidate or ketamine was administered in a randomized, crossover fashion (2 or 4 mg/kg and 20 or 40 mg/kg, respectively) on eight consecutive days. Following recovery of righting reflex, animals were assessed for residual effects on functional mobility on the balance beam, motor behavior in the open field and adrenal function through ACTH stimulation. We evaluated the consequences of the effects of the anesthetic agent-induced motor behavior on functional mobility. On the balance beam, etomidate-treated rats maintained their grip longer than ketamine-treated rats, indicating greater balance abilities (mean ± SD, 21.5 ± 25.1 s *vs.* 3.0 ± 4.3 s respectively, *p* < 0.021). In the open field test, both dosages of etomidate and ketamine had opposite effects on travel behavior, showing ketamine-induced hyperlocomotion and etomidate-induced hypolocomotion. There was a significant interaction between anesthetic agent and motor behavior effects for functional mobility effects (*p* < 0.001). Corticosterone levels were lower after both 40 mg/kg ketamine and 4 mg/kg etomidate anesthesia compared to placebo, an effect stronger with etomidate than ketamine (*p* < 0.001). Following recovery from anesthesia, etomidate and ketamine have substantial side effects. Ketamine-induced hyperlocomotion with 20 and 40 mg/kg has stronger effects on functional mobility than etomidate-induced hypolocomotion with 2 and 4 mg/kg. Etomidate (4 mg/kg) has stronger adrenal suppression effects than ketamine (40 mg/kg).

## 1. Introduction

More than 21 million patients undergo general anesthesia in North America each year [1]. Perioperatively, anesthetic agents are administered to ensure loss of consciousness and to provide sufficient analgesia and muscular relaxation during surgery. Moreover, single boluses of both etomidate and ketamine are used during procedures of short duration, such as electroconvulsive therapy (ECT) [2], as well as for emergency intubations in the emergency room or intensive care unit [3].

All anesthetics have the ability to depress brain stem neuronal activity, which can result in apnea and severe arterial hypotension [4]. Therefore, careful monitoring is obligatory, until sufficient breathing, functional mobility and cognitive functioning are secured [5].

Little is known regarding anesthetic effects after the return of consciousness. In order to identify the anesthetic that can provide unconsciousness and have the ability to allow rapid recovery of brain function, post-awakening pharmacological effects of these agents need to be assessed. In this study, we evaluated the residual effects of etomidate and ketamine on functional mobility and adrenocortical hormone production, so that the consequences of these anesthetics’ effects can be assessed and optimally monitored in patients after surgery. We aimed to assess the effects of anesthetic agent-induced motor behavior disturbance on functional mobility impairment after emergence from anesthesia.

We hypothesized that, at anesthetic levels after the recovery of righting reflex,

1 residual etomidate and ketamine would impair functional mobility and motor behavior,
2 residual ketamine would modify anxiety-related behavior, and
3 residual etomidate would impair adrenal function.

## 2. Experimental Section

### 2.1. Animals

The study was conducted in accordance with the rules and regulations of the Institutional Animal Care and Use Committee at Massachusetts General Hospital, Boston (Protocol 2011N00181). A total of 24 adult male Sprague–Dawley rats (240–500 g) were purchased from Charles River Laboratories (Wilmington, MA) and housed in the Massachusetts General Hospital Center for Comparative Medicine animal care facility. All animals were acclimated to the experimental environment for 4 days before any testing.

### 2.2. Instrumentation

All animals were anesthetized (5% isoflurane and maintained with 1.5% isoflurane) and instrumented with a lateral tail vein intravenous catheter (24 gauge, 19 mm). A rectal probe was inserted for continuous monitoring of body temperature in order to maintain a temperature of 36.5 °C with the aid of a heating pad.

### 2.3. Evaluation of Test Drug Effects on Animal Behavior

#### 2.3.1. Anesthetic Intervention and Righting Reflex Recovery

**Figure 1:**
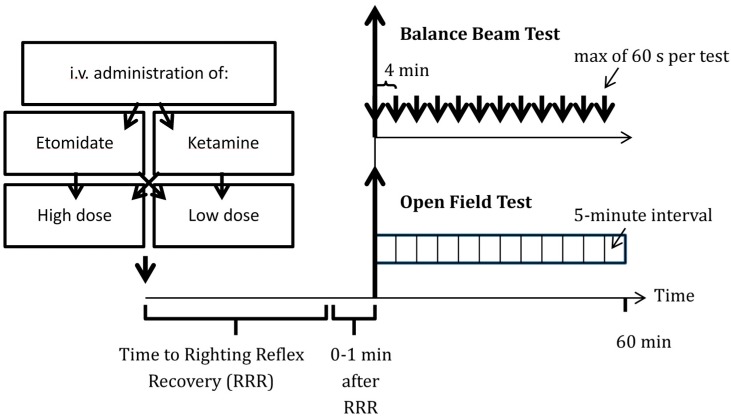
Protocol used to evaluate etomidate’s and ketamine’s residual effects on functional mobility, motor behavior and anxiety-related behavior. Rats received i.v. ketamine or etomidate in high or low doses (20 or 40 mg/kg ketamine *vs.* 2 or 4 mg/kg etomidate) in a cross-over fashion. Following drug administration, the time to recovery of righting reflex was recorded, followed by balance beam and open field tests at 4- and 5-minute intervals, respectively.

Six rats received a single intravenous bolus of either (1) 20 mg/kg ketamine, (2) 40 mg/kg ketamine, (3) 2 mg/kg etomidate or (4) 4 mg/kg etomidate on eight consecutive days, in a randomized fashion in order to minimize order effects (Figure 1). Dosages were chosen based on previous studies, titrated to arrive at equipotency for the endpoint duration of action. The animal was placed in a clear rat restrainer tube in which it was supplied with O_2_ at 2 L/min, and the initial isoflurane anesthesia was stopped. The test drug was administered according to the randomization as soon as the rat regained consciousness and righting reflex. Following drug administration, the rats were placed in the supine position and were left without a heating pad in order to record the time to recovery of righting reflex (RRR). This period is defined as the time from drug administration to the return to upright position with all four paws oriented towards the ground [6].

#### 2.3.2. Post-Anesthetic Behavioral Testing

Within 1 min of recovery of righting reflex, the animals were randomized to either the balance beam test (BBT) for evaluation of functional mobility or the open field test (OFT) for evaluation of motor behavior and anxiety-related behavior (Figure 1). After a 24-hour recovery, the protocol was repeated daily and at the same time, until every animal completed all eight conditions (2 or 4 mg/kg etomidate or 20 or 40 mg/kg ketamine followed by OFT or BBT). The same group of investigators conducted all behavioral experiments with the same activity distribution throughout all experiments [7]. All equipment was wiped with 70% ethanol between animals in order to erase olfactory stimuli.

##### 2.3.2.1. Balance Beam Test

The animals were encouraged to cross over a narrow wooden beam (80 cm × 2.5 cm × 2.5 cm) into a darkened goal box at the opposite end, 60 cm over the ground [8,9]. As described by Dixon *et al.*, noxious stimuli (bright light and high decibel white noise) were applied and terminated once entering the box. The animals were tested 12 times for a maximum of 60 s every 4 min (Figure 1) [8]. All animals were previously trained in 3 independent training sessions one day before the first testing day, and baseline measurements were conducted on the day of first testing. A Sony DCR-SX85 Handycam Camcorder recorded all testing. The primary aim of our balance beam testing was to assess the stepwise recovery of functional mobility after emergence of anesthesia. In order to describe the recovery, a blinded observer subsequently assessed whether the rat was able or unable to cross the beam in each session. We also quantified the time the animal was able to hold its body on the beam (in a scenario where the rat was unable to cross the beam) or the time it took to cross the beam (in a scenario where the beam could be crossed). We describe functional mobility with 3 different measures: balancing time in absence of crossing ability, time to recovery of crossing ability and the crossing time in presence of crossing ability.

##### 2.3.2.2. Open Field Test

Within the first minute after recovery of righting reflex, the rats were placed in the center of an open field (OF) for a total time of 60 min. The open field was 100 cm × 100 cm × 40 cm, and the center was defined as the 9 quadrants in the middle of the apparatus and the margin zone as the surrounding 16 quadrants [10,11]. Testing was conducted in 5 min intervals, as previously described (Figure 1) [12]. Following the experiment, endpoints were measured every 5 min using the Any-maze video-tracking software version 4.5 (Stoelting Co., Wood Dale, IL, USA), and include: (1) motor behavior (assessed as the distance traveled); and (2) anxiety-related behavior (assessed as the number of entries in the central zone). On the day before testing, each rat went through a 30-minute habituation period during which baseline measurements were taken (Figure 1) [10].

#### 2.3.3. Adrenocortical Function Testing

**Figure 2:**
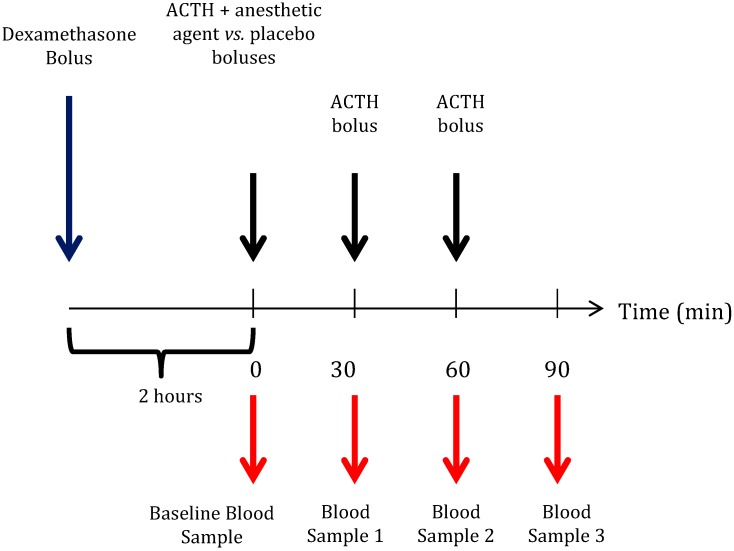
Protocol used to evaluate etomidate’s and ketamine’s residual effects on adrenal function. Two hours following dexamethasone bolus administration (0.2 mg/kg), a baseline blood sample was drawn prior to the administration of the first dose of adrenocorticotropic hormone 1–24 (ACTH (1–24)) (25 µg/kg). Then, a bolus of high dose ketamine (n = 6), etomidate (n = 6; 40 mg/kg *vs.* 4 mg/kg) or placebo (n = 6) was administered in a parallel group design. At 30-minute intervals, two further doses of ACTH were administered, and blood samples were taken.

To evaluate the differential effects of etomidate and ketamine on adrenal function, 18 additional rats were tested in a parallel group fashion based on a protocol by Cotten *et al.* (etomidate, n = 6 *vs.* ketamine, n = 6 *vs.* placebo, n = 6, Figure 2) [13]. Each rat was given dexamethasone (0.2 mg/kg intravenously) at the beginning of each experiment to suppress the physiological stimulation of the adrenocortical hormone production by adrenocorticotropic hormone 1-24 (ACTH (1-24)). During the subsequent 2 h, the rats were allowed to rest in their cages before they were re-anesthetized with isoflurane, as previously described.

Before starting the experimental protocol, a blood sample was drawn to determine the un-stimulated baseline plasma corticosterone concentration. Subsequently, a second dose of dexamethasone was administered to keep the physiological ACTH production of the animal suppressed. High dose etomidate (4 mg/kg), ketamine (40 mg/kg) or placebo was then administered as a bolus. Adrenocortical function (*i.e.*, responsiveness to ACTH (1–24) administration) was assessed by repetitive administration of ACTH (1–24) (25 µg/kg intravenously) and by measuring plasma corticosterone concentrations 30 min later. The first dose of ACTH (1–24) was given directly before the test drug administration, and the plasma corticosterone concentration was measured 30 min later (Blood Sample 1 in Figure 2). Immediately after drawing Blood Sample 1, a second dose of ACTH (1–24) was given, and the plasma corticosterone concentration was measured 30 min later (Blood Sample 2 in Figure 2). The same procedure was repeated a third time in order to obtain Blood Sample 3 (Figure 2).

The volume of each blood sample was approximately 0.3 mL. The plasma corticosterone concentrations were determined as previously reported [14]. Blood samples were centrifuged at 10000 rpm for 10 min at 4 °C, and the supernatant plasma was collected. The samples were frozen (−80 °C) pending corticosterone measurement.

After thawing and heat inactivation of corticosterone binding globulins (65 °C for 20 min), plasma baseline and ACTH (1–24) -stimulated corticosterone concentrations were quantified using the enzyme-linked immunosorbent assay (Cayman Chemicals, Ann Arbor, MI) and a 96-well plate reader (Molecular Devices, Sunnyvale, CA, USA).

### 2.4. Statistical Analysis

The primary aim of this study was to evaluate the residual effects of 2 and 4 mg/kg etomidate and 20 and 40 mg/kg ketamine on functional mobility on the balance beam and on motor behavior in the open field. Secondarily, we determined the effects of 20 and 40 mg/kg ketamine on anxiety-related behavior after recovery of consciousness following anesthesia. We also analyzed the differential effects of 4 mg/kg etomidate and 40 mg/kg ketamine on the adrenocortical hormone production.

In order to address the primary aim of the study, we tested the hypothesis: (A) that the two anesthetic agents impair functional mobility (primary outcome) and motor behavior (secondary outcome); and (B) that 2 and 4 mg/kg etomidate, as well as 20 and 40 mg/kg ketamine-induced disturbance of functional mobility and motor behavior differ in quality. We included all measurements of motor function assessment using a mixed linear model (compound symmetry repeated covariance type) with an identity link function for normally-distributed probability.

To evaluate the effects of anesthesia on the primary outcome, we included anesthetic agent (baseline *vs.* ketamine *vs.* etomidate), dose (low *vs.* high) and time interval (interval 1–12) as repeated independent variables and “crossing time on the beam” as the dependent variable. We tested for the main effect of the anesthetic agent. The same model was used to evaluate the effects on motor behavior, using the “mean distance traveled within 5-minute intervals in the OF” as the dependent variable.

To assess the differences in quality of functional mobility impairment and motor behavior, we used the above-mentioned model and tested for an interaction effect of each anesthetic agent over time on the dependent variables “time maintaining grip” on the beam and “mean distance traveled” in the OF.

In an exploratory analysis, we investigated the effects of anesthetic-induced changes in motor behavior on functional mobility. We used the same mixed model as for the primary outcome and tested for an interaction effect of anesthetic compound and the absolute difference in distance traveled on the dependent variable “time maintaining grip” on the beam.

We addressed the second aim by testing the hypothesis that ketamine after recovery of consciousness following anesthesia affects the anxiety behavior of animals, expressed as entries into the central zone of the open field. We tested for an effect of 20 and 40 mg/kg ketamine over time compared to baseline and for an interaction between the slope of both dosages and the time on the number of entries into the central zone.

For our third aim, we hypothesized that 4 mg/kg of etomidate and 40 mg/kg of ketamine affect adrenocortical hormone production. A one-way ANOVA with Bonferroni correction was used to test the difference in mean stimulated corticosterone plasma concentration.

All data are reported as the means ± SD, unless otherwise specified. Statistical analysis was performed using SPSS 22.0 (SPSS Inc., Chicago, IL, USA).

## 3. Results

A total of 48 behavioral tests were conducted in six rats on eight consecutive days. The adrenocortical function tests were performed in 18 rats.

**Figure 3:**
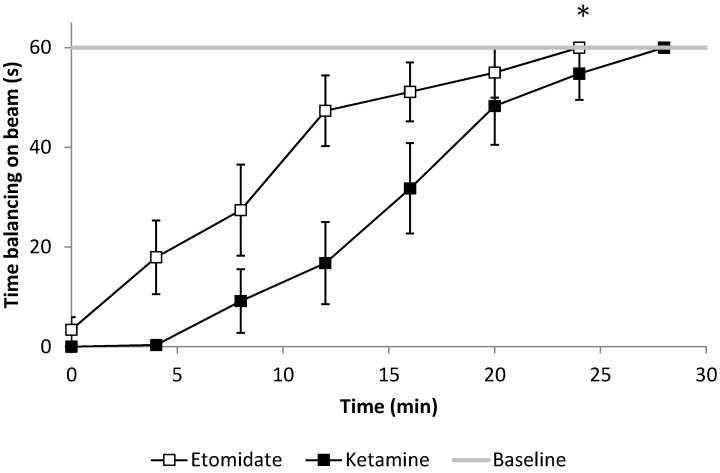
Functional mobility described as the ability to maintain balance on the beam, assessed every four minutes after emergence. After 2 and 4 mg/kg etomidate anesthesia, rats were able to hold themselves significantly longer on the beam compared to 20 and 40 mg/kg ketamine (* *p* = 0.021 for the slope of the gripping time comparison over time, n = 6). Error bars represent the SEM.

Rats treated with 2 mg/kg etomidate recovered faster from loss of righting reflex than those treated with 4 mg/kg etomidate (14.3 ± 6.6 min *vs.* 26.1 ± 8.9 min, *p* < 0.001, Figure 3). Likewise, rats that received 20 mg/kg ketamine recovered faster than those with 40 mg/kg ketamine (7.8 ± 2.1 min *vs.* 25.4 ± 6.1 min, *p* < 0.001). The mean recovery time did not differ significantly between the two anesthetic agents (*p* = 0.201).

### 3.1. Primary Aim: Residual Effects of Etomidate and Ketamine on Functional Mobility and Motor Behavior

Etomidate (2 and 4 mg/kg) and ketamine (20 and 40 mg/kg) led to significantly impaired functional mobility, as quantified by longer crossing times on the balance beam (*p* < 0.021) and significantly impaired motor behavior in the open field test (*p* < 0.001) compared to baseline.

While rats treated with 20 and 40 mg/kg of ketamine and 2 and 4 mg/kg of etomidate needed the same time to recover their ability to cross the beam (pooled data for etomidate vs. ketamine, 35.6 ± 13.7 s *vs.* 35.8 ± 11.0 s, *p* = 0.976), animals maintained balance significantly longer on the beam following etomidate compared to ketamine anesthesia (21.5 ± 25.1 s *vs.* 3.0 ± 4.3 s respectively, *p* < 0.021, Figure 3), in a dose-dependent manner (*p* = 0.049 for the etomidate x dose interaction).

**Figure 4:**
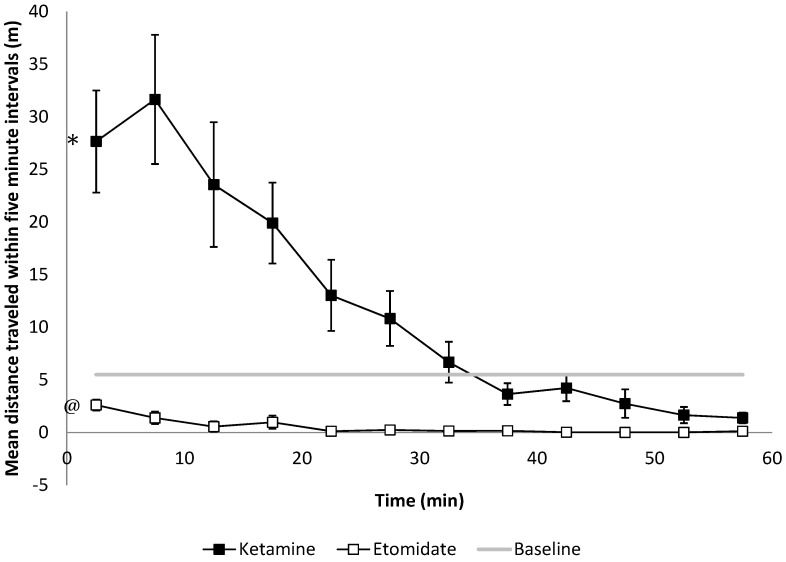
Motor function as assessed by spontaneous travel behavior in the open field evaluated within 5-min intervals. After 20 and 40 mg/kg of ketamine treatment, rats travel significantly longer distances compared to baseline (* *p* < 0.001 the slope of the distance travelled compared to the baseline, n = 6), while 2 and 4 mg/kg of etomidate anesthetized rats travel significant shorter distances within every 5-minute interval compared to the baseline (@ *p* = 0.009, n = 6). Error bars represent the SEM.

We observed opposite effects of 2 and 4 mg/kg of etomidate (decreasing) and 20 and 40 mg/kg of ketamine (increasing) on spontaneous motor behavior in the OF. The mean distance travelled within every 5-min interval after both doses of etomidate *versus* both doses of ketamine amounted to 0.5 ± 1.3 m and 12.2 ± 14.61 m, respectively compared with baseline 5.5 ± 6.33 m (*p* < 0.01, Figure 4).

The differential anesthetic effects on motor behavior were found to be an independent predictor of functional mobility with higher absolute differences in distance travelled in the open field, resulting in significantly worse functional mobility (*p* < 0.001 for the interaction of anesthetic agent and absolute difference in distance travelled in the open field on ‘time maintaining grip’ on the balance beam).

### 3.2. Secondary Aim: Effects of Ketamine on Anxiety-Related Behavior

**Figure 5:**
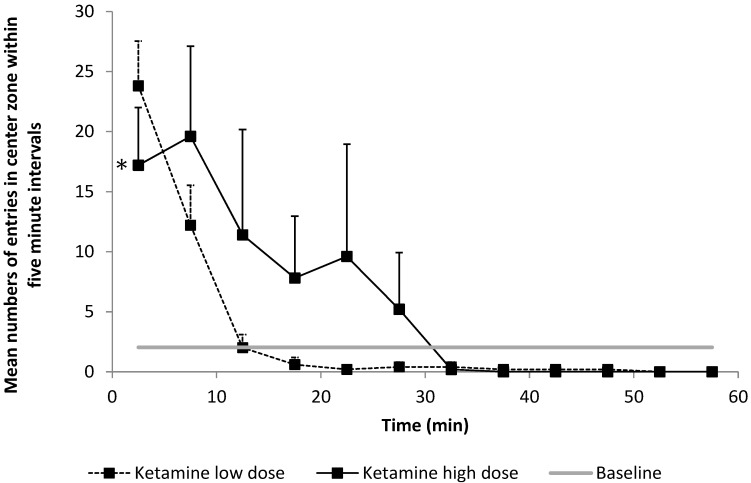
Number of entries into the central zone indicating the anxiety-related behavior of the animal, as evaluated within 5-min intervals. Compared to baseline, rats entered the central zone of the open field (OF) more frequently after ketamine anesthesia (*p* < 0.021). This effect was shown to be significantly higher after 40 mg/kg ketamine *vs.* 20 mg/kg ketamine (* *p* = 0.049).

Residual ketamine anesthesia after injection of 20 mg/kg and 40 mg/kg resulted in a significantly higher frequency of entries into the central zone of the OF compared to the baseline (4.63 *vs.* 2.03, respectively, *p* < 0.001 for the slope of ketamine over time compared to the baseline). We observed a dose-dependent effect with a higher frequency of entries into the central zone with higher dosages (5.9 ± 12.1 *vs.* 3.4 ± 7.7, *p* < 0.001, Figure 5).

### 3.3 Third Aim: Differential Effects of Etomidate and Ketamine on the Adrenocortical Hormone Production

**Figure 6:**
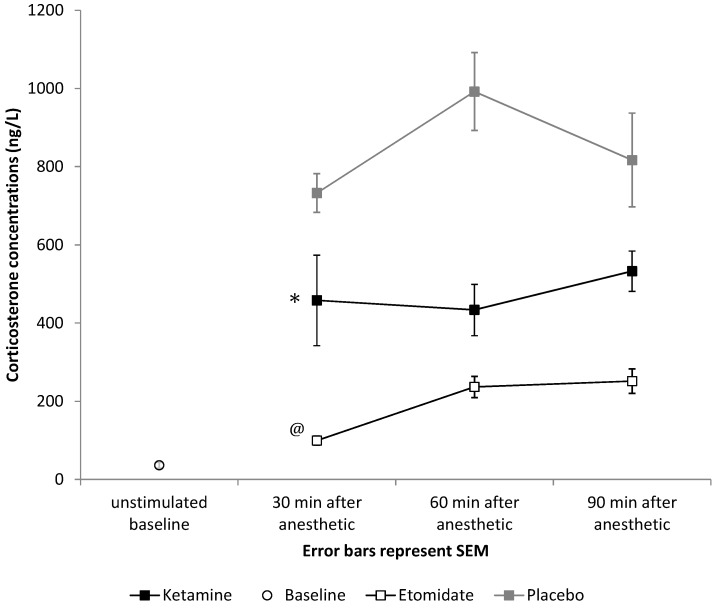
Effects of a bolus of 4 mg/kg of etomidate or 40 mg/kg of ketamine on free plasma corticosterone concentrations compared to placebo (saline bolus). After both 4 mg/kg of etomidate and 40 mg/kg of ketamine, significantly lower responses to ACTH were observed during the full study period (* *p* < 0.001 for mean ACTH response after ketamine compared to placebo, and @ *p* < 0.001 for mean ACTH response after etomidate compared to placebo). Error bars represent the SEM.

Baseline plasma corticosterone concentration was 36.3 ± 63.4 ng/mL following dexamethasone treatment. Significantly lower corticosterone levels following ACTH administration were observed in the 4 mg/kg of etomidate group compared to controls (192.4 ± 119.5 ng/mL *vs.* 847.7 ± 400.1 ng/mL, *p* < 0.001, Figure 6).

Plasma corticosterone levels in the ketamine group were also significantly lower compared to the control group (472.1 ± 318.9 ng/mL *vs.* 847.7 ± 400.1 ng/mL, *p* < 0.001; Figure 6), yet significantly higher than in the etomidate group (472.1 ± 318.9 ng/mL *vs.* 192.4 ± 119.5 ng/mL, *p* < 0.001; Figure 6). We did not find an effect of order on any of our behavioral endpoints (righting reflex recovery, balancing time, crossing time, delta in distance travelled, distance travelled, entries into the central zone).

## 4. Discussion

### 4.1. Summary of Findings

In this prospective, randomized controlled interaction study in rats, we found that ketamine and etomidate after recovery of righting reflex impair functional mobility. However, after recovery of consciousness following anesthesia, 20 and 40 mg/kg of ketamine led to hyperlocomotion, while 2 and 4 mg/kg of etomidate induced hypolocomotion. Both 40 mg/kg of ketamine and 4 mg/kg of etomidate suppress the production of adrenocortical hormones, but the effect is stronger with 4 mg/kg of etomidate.

The dosages of the two drugs chosen for this experiment showed equipotency on the endpoint ‘time to righting reflex recovery’, indicating similar effects of time to awakening after general anesthesia [6]. Based on the comparability of dosages, we are able to directly compare the quality and quantity of the residual effects of both anesthetics after awakening.

Lingering effects of anesthetics translate to increased morbidity and costs. In our study, we observed that both ketamine and etomidate induced an impairment of functional mobility after emergence, which requires precautions for patients’ safety. Single boluses of both etomidate and ketamine are used during procedures of short duration, such as electroconvulsive therapy (ECT) [2], as well as for emergency intubations in the emergency room or intensive care unit [3]. Thus, we believe that our study design translates to perioperative medicine. Impairment of functional mobility after anesthesia puts the patient at risk of falling [15]. In clinical anesthesia, the minimal requirements for safe recovery and discharge include the ability of patients to dress themselves and to walk out [16]. Even after being under anesthesia for a short period only, patients are asked to refrain from driving or operating hazardous machinery for at least 24 h due to the residual effects of the anesthetics [17].

In order to evaluate the residual effects of anesthesia on functional mobility, we used the balance beam test as a model [18,19]. We believe that testing coordination and balance of rodents after anesthesia on the balance beam translates to the postoperative functional mobility of patients, including (complex vestibulomotor) activities, such as walking, driving and operating machines. Furthermore, we believe that spontaneous motor behavior as assessed in the OF could reflect the activity level of patients post-emergence due to the residual effects of anesthesia. In patients, the Richmond Agitation and Sedation Scale (RASS) can help as a valuable tool to identify the motor behavior levels between agitation and sedation [20].

### 4.2. Hyperlocomotion and Hypolocomotion both Translate into Impaired Functional Mobility

During emergence from anesthesia, patients may react with symptoms of sedation and a certain level of “hypoactivity” or agitation, coupled with “hyperactivity”. In our experiments, after administration of 2 and 4 mg/kg of etomidate, rats showed hypolocomotion as assessed by reduced travel behavior during the entire post-anesthetic observation period of 60 min [21]. Following 20 and 40 mg/kg of ketamine, animals demonstrated hyperlocomotion by traveling significantly longer distances compared to the baseline during the first 30 min of the observation period.

The differential effects of ketamine and etomidate on motor behavior translated significantly to impaired functional mobility independent of quality. In our experiments, ketamine-anesthetized rats also demonstrated changes in behavioral patterns. The natural behavior of rats placed in an open field is to remain in close contact with the walls, a behavior that is termed thigmotaxis: the animals appear to avoid the unknown and potentially dangerous open area. An anxiolytic effect is assumed if a rat spends less time in close proximity to the walls instead and increases exploratory activity in the central compartment [22,23,24]. Our rats showed risky behavior after emergence from 20 and 40 mg/kg of ketamine as assessed by the higher incidence of entries in the central zone of the open field. Both hyperactivity and reduced anxiety of animals could be classified as delirium-like behavior after ketamine administration [25,26,27].

Following emergence from ketamine anesthesia, patients go through a dissociative state in which they perceive nociceptive stimuli, but no pain, due to the disruption of cortical integration, preventing pain perception [27,28,29]. During recovery from anesthesia, auditory and visual hallucinations, as well as increased motor activity are frequently reported [30]. This clinical picture has been defined as ketamine-induced delirium [31,32]. Ketamine’s principal molecular target is the NMDA receptor, a major postsynaptic, ionotropic receptor for the excitatory neurotransmitter, glutamate [33]. In contrast, etomidate acts as a GABA_A_ receptor agonist. GABA_A_ receptors are widely distributed throughout the brain [34]. Binding of a GABA_A_ hypnotic to the GABA receptors helps maintain postsynaptic chloride channels in the open position, thereby enhancing the inward chloride current, which hyperpolarizes the pyramidal interneurons [35]. Because small numbers of inhibitory interneurons control large numbers of excitatory pyramidal neurons, hypnotic-induced enhancement of GABA_A_ inhibition can efficiently inactivate large brain regions [36]. The clinical signs are consistent with inhibitory actions in the brainstem, leaving the patients with residual sedation after emergence from anesthesia.

Hyperlocomotion and hypolocomotion in our rats resulted in impaired functional mobility after emergence. Interestingly, in our study, etomidate-induced hypolocomotion with 2 and 4 mg/kg was associated with a slightly better functional mobility compared to ketamine-induced hyperlocomotion with 20 and 40 mg/kg. Clinicians need to consider that both hypoactive and hyperactive states translate into an impairment of functional mobility. Traditionally, we focus, in the setting of perioperative medicine, on the exclusion of hypoactive states and impairment of arousal. In the post anesthesia care unit (PACU), we typically assess reduced levels of activity and consciousness by means of the Aldrete Scoring System, which has become an integral part of discharge criteria after anesthesia [37]. However, we do not routinely assess for hyperactivity as a symptom of impaired functional mobility.

Our data suggest that we may apply tests that also capture hyperactive states and delirium. We have recently started using tests, such as the Confusion Assessment Method for the Intensive Care Unit (CAM-ICU) and might consider delivering those tools also to the PACU [38].

### 4.3. Ketamine Suppresses Adrenal Function

The assessment of adrenocortical function revealed suppressed steroidogenesis for both 40 mg/kg of ketamine and 4 mg/kg of etomidate. Etomidate is well known to inhibit 11-beta-hydroxylase, a cytochrome P450 enzyme necessary for corticosterone, cortisol and aldosterone synthesis [39,40,41]. It causes adrenal insufficiency that lasts more than 24 h after drug administration [42]. Based on the higher morbidity and mortality associated with this effect [43,44,45], it is barely used as an infusion [46].

This is the first time that an isolated suppressant effect of ketamine on the adrenocortical hormone production has been described. In a multicenter randomized clinical trial, Jabre *et al.* evaluated the effects of ketamine and etomidate used for emergency endotracheal intubation in critically ill patients. Using an ACTH-stimulation test, the authors found that after ketamine administration, 48% of patients showed adrenal insufficiency, defined as baseline cortisol of less than 276 nmol/L, or the maximum change (peak cortisol minus baseline cortisol) of less than 250 nmol/L, or both. Consistent with our findings, the authors described a significantly higher decrease of the cortisol level after etomidate anesthesia compared with ketamine anesthesia, resulting in adrenal insufficiency in 86% of patients receiving etomidate *vs.* 48% of patients receiving ketamine. However, the high incidence of adrenal failure in patients given ketamine was not attributed to substance-specific effects, but to critical illness [3].

A mechanism similar to etomidate, which inhibits the CYPIIB enzymes by binding with their imidazole ring to the CYPIIB heme iron, is unlikely, since ketamine does not contain such a structure [47]. However, independent predictors of low cortisol response to adrenocorticotropin hormone stimulation include very low arterial blood pH, bicarbonate or platelet count, as well as high disease severity and organ failure [48]. Most of these factors should not affect the results of our study, since all study animals were young and healthy. However, an unspecific respiratory depressant effect of general anesthesia leading to decreased pH and high bicarbonate blood levels could have led to the observed reduced corticosterone response [49].

## 5. Conclusions

Following recovery from 2 and 4 mg/kg of etomidate and 20 and 40 mg/kg of ketamine, we observed substantial side effects in rats. Ketamine-induced hyperlocomotion after intravenous injection of 20 and 40 mg/kg has stronger effects on functional mobility than etomidate-induced hypolocomotion after injection of 2 and 4 mg/kg. Etomidate (4 mg/kg) has stronger adrenal suppression effects than ketamine (40 mg/kg).

Hyperactive states and delirium might need to be assessed in patients in the PACU. Recently introduced tests in intensive care units, such as the Confusion Assessment Method for the Intensive Care Unit (CAM-ICU), could be considered for this assessment.

The effects of ketamine causing adrenal insufficiency will need to be studied in a dedicated trial in critically ill patients.

## 6. Limitations

The anxiolytic effects of etomidate are methodologically difficult to assess in the current model, because etomidate-induced anxiolytic behavior cannot be demonstrated in an animal that still shows some signs of immobility as a consequence of lingering etomidate effects. However, this does not affect our primary aim, which was assessing the effects of anesthetic agent-induced hyper- and hypo-locomotion on functional mobility impairment after emergence from anesthesia.
